# Constrained acetabular liners in total hip arthroplasty: analysis of 265 primary and revision cases from the Dutch Arthroplasty Register (2007–2022)

**DOI:** 10.1177/11207000251331147

**Published:** 2025-04-01

**Authors:** Jetze Visser, Mirthe H W van Veghel, Liza N van Steenbergen, Bart A Swierstra, Esther M Bloemheuvel, B Willem Schreurs

**Affiliations:** 1 Department of Orthopaedics, Radboud University Medical Centre, Nijmegen, the Netherlands; 2 Dutch Arthroplasty Register (LROI), ‘s Hertogenbosch, the Netherlands

**Keywords:** Constrained acetabular liner, total hip arthroplasty, dislocation, registry data

## Abstract

**Introduction:**

Constrained acetabular liners (CALs) are predominantly used as a salvage procedure in patients with a severe risk for dislocation after total hip arthroplasty (THA). However, the constrained design of CALs bears the risk of impingement with subsequent loosening or failure of the integrity of the implant. We investigated the use and survival of CALs in primary and revision THA in the Dutch Arthroplasty Register (LROI).

**Methods:**

Use of CAL in THAs was extracted from the LROI in the period 2007–2022. 423,773 primary THAs and 52,706 revision THAs have been registered, of which 29 CALs were implanted in primary THA and 236 CALs in revision THA. Patient characteristics and survival of the CAL placed in primary and revision THA were separately analysed.

**Results:**

Of the CALs placed in primary THA, no revisions of the implant occurred during a median follow-up of 5.4 years (interquartile range [IQR] 4.0–12.4). The CALs in revision THA were most frequently used for cases with recurrent dislocation (183/236). Median follow-up was 4.8 (IQR 2.3–8.2) years. The re-revision rate was 10% (95% CI, 6.6–14) at 5-year follow-up and 12% (CI, 8.1–17) at 9-year follow-up. The most frequently registered reason for re-revision was dislocation (*n* = 19, 70%).

**Conclusions:**

In the Netherlands there is a relatively low use of CALs in primary as well as revision THA. The survival rate of CALs is acceptable, with recurrent dislocation as the main reason for re-revision. The use of CALs should be reserved for specific cases with high risk for dislocation.

## Introduction

The risk of dislocation following primary total hip arthroplasty (THA) is decreasing due to advances in surgical approach and implant designs.^1^ The key improvements in prosthesis design have been the use of larger diameter femoral heads and dual-mobility bearings.^2,3^ Nevertheless, dislocation is still a significant complication of THA, which leads to inferior outcomes for the patient and comes with the risk of recurrent instability and subsequent revision surgery.^4^ Recurrent instability can occur because of patient characteristics, such as abnormal anatomy of the pelvis, stiffness of the spine, abductor insufficiency, or obesity. Also, a suboptimal or even incorrect placement of the implants plays an important role. Unfortunately, when revision surgery for dislocation is required, the risk of re-dislocation still remains relatively high.^4^ To this end, constrained acetabular liners (CAL) have been designed, in which the femoral head is captured in the acetabular component, hence reducing the risk of dislocation.^5^ However, CALs are associated with a reduced range of motion of the hip joint by the nature of their constrained design.^6^ As a result, impingement is the primary mechanism of failure of these implants, potentially leading to failure of the constrained system with subsequent dislocation, intra-component failure or loosening of the acetabular component. CALs are therefore predominantly used as a salvage procedure in patients with a severe risk for dislocation, such as abductor insufficiency, a neuromuscular disorder or recurrent dislocations despite previous revision surgery.^7^ A recently published systematic review of the survival of CALs placed in primary and revision THA found a reoperation-free survivorship of 79.9% after 6.9 years.^7^ We found only 1 previous register study on CALs in primary THA showing an 8-year survivorship for any reason of 94%.^8^ Our study is the first register study to investigate patient characteristics, survival and reasons for revision of CALs implanted in primary and revision THA.

## Methods

The LROI (Landelijke Registratie Orthopedische Interventies, Dutch Arthroplasty Register) is a nationwide population-based register that has included information on primary and revision arthroplasties in the Netherlands since 2007. Since 2012 all Dutch care providers report to the LROI.^9^ The completeness of reporting is over 99% for primary THAs and 97% for hip revision arthroplasty.^10^

The LROI database contains information on patient, procedure, and prosthesis characteristics. In 2014, body mass index (BMI) and smoking were added to the LROI database. For each component, a product number is registered to identify the characteristics of the prosthesis. The vital signs status of all patients is obtained actively on a regular basis from Vektis, the national insurance database on health care in the Netherlands, which records all deaths of Dutch citizens. The LROI uses the opt-out system to require informed consent of patients.

For the current study, we included THAs from the LROI where a CAL was used in the period 2007–2022. The procedures were divided into a cohort that received a CAL in primary THA (*n* = 29) and a cohort that received a CAL in hip revision surgery (*n* = 236). CALs were registered in the LROI as a cup or as a liner. Some CALs were clicked into an uncemented cup, while other liners were cemented in a pre-existing uncemented metal shell. Patient characteristics, surgical approach, type of implant fixation and articulation were reported. Revision is defined as a replacement, removal or addition of one or more components of the prosthesis. Closed reduction after a dislocation, open reduction without component exchanges or incision and drainage for infection are not included in the LROI.

### Statistics

Descriptive statistics were used to summarise patient, prosthesis, and procedure characteristics of the primary and revision THAs. Cumulative crude incidence including 95% confidence intervals (CI) of overall re-revision was calculated in the revision cohort using competing risk analysis, where death was considered to be a competing risk.^11,12^ Survival was calculated as the time from implantation of the CAL during revision arthroplasty to the first re-revision arthroplasty for any reason, death of the patient, or the end of the study follow-up (01 January 2023). In the revision cohort, the type of revision and reasons for revision in which the CAL was placed were described, as well as the type of re-revision and reasons for re-revision.

The dataset was processed in compliance with the regulations of the LROI governing research on registry data. No external funding was received. No competing interests were declared. This study was reported in accordance with the STROBE guidelines.

## Results

Between 2007 and 2022, 423,773 primary THAs and 52,706 revision THAs were registered in the LROI. In 29 primary THAs a CAL was used, of which 14 patients were ASA III–IV. The mean age of the patients was 71 (standard deviation [SD] 12) years and 11 procedures were performed in patients who had had previous surgery on the affected hip. 24 CALs were used with cementless fixation (Table 1). In revision THA, 236 procedures were registered, of which 166 (70%) were carried out with cementless fixation (Table 2). The patients in this revision cohort had a mean age of 76 (SD 11) years and 87 patients were ASA III–IV.

**Table 1.** Patient, procedure and prosthesis characteristics of primary THAs with constrained acetabular liners.

Patient, procedure and prosthesis characteristics	*n* = 29
Male sex	8
Age (mean (SD))	71 (12)
ASA score	
ASA I	3
ASA II	12
ASA III-IV	14
Body mass index	
Underweight (⩽18.5)	1
Normal weight (>18.5-25)	12
Overweight (>25-30)	3
Obesity (>30-40)	1
Before 2014: not registered	12
Smoking (%)	2
Before 2014: not registered	12
Previous surgery at affected hip	11
Fixation	
Cemented	4
Cementless	24
Hybrid (femur cemented)	1
Surgical approach	
Anterior	3
Posterolateral	25
Straight lateral	1
Articulation	
Ceramic-on-polyethylene	12
Metal-on-polyethylene	17
Femoral head size	
28 mm	7
32 mm	13
36 mm	2
Missing	7

ASA, American Society of Anesthesiologists.

**Table 2.** Patient and prosthesis characteristics of revision THAs with constrained acetabular liners.

Patient and prosthesis characteristics	N = 236
Male sex (%)	61 (25)
Age (mean (SD)) (%)	76 (11)
ASA score (%)	
ASA I	10 (4.2)
ASA II	127 (54)
ASA III-IV	87 (37)
Missing	12 ( 5.1)
Body mass index (%)	
Underweight (⩽18.5)	7 ( 3.0)
Normal weight (>18.5-25)	65 (27)
Overweight (>25-30)	50 (21)
Obesity (>30-40)	22 ( 9.3)
Morbid obesity (>40)	3 (1.3)
Before 2014: not registered	89 (37)
Smoking (%)	20 ( 8.5)
Before 2014: not registered	89 (37)
Missing	3 (1.3)
Fixation (%)	
Cemented	56 (23)
Cementless	166 (70)
Hybrid	10 (4.2)
Missing	4 (1.7)
Femoral head size (%)	
22 mm	24 (10)
28 mm	46 (19)
32 mm	66 (28)
36 mm	26 (11)
Missing	74 (31)

ASA, American Society of Anesthesiologists.

No revisions of the THA with CAL in primary surgery occurred during a median follow-up of 5.4 years (interquartile range [IQR] 4.0–12.4). 5 patients died during follow-up.

**Figure 1.**
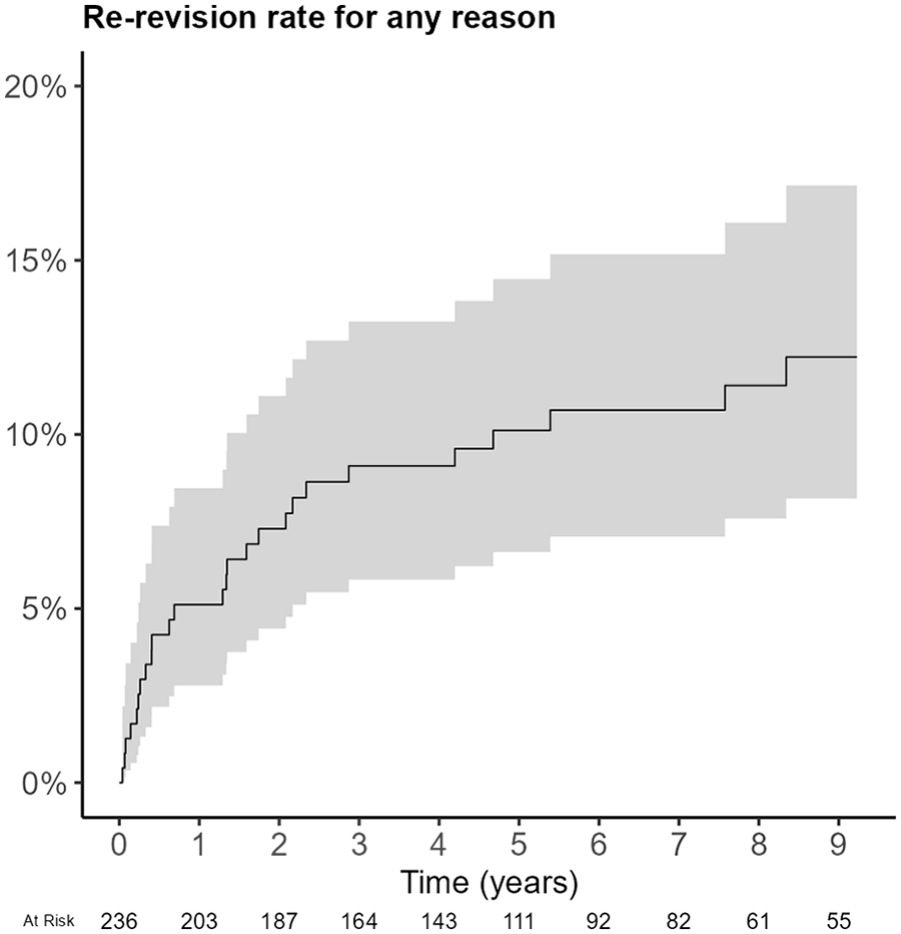
Competing risk analysis of re-revision of the constrained acetabular liners implanted in revision surgery.

In THAs in which a CAL was placed in revision surgery, recurrent dislocation was the most frequent reason for revision (*n* = 183, 81%) (Table 3). In 123 (52%) revision procedures at least the acetabular component was revised. In 79 (34%) procedures the femoral head and/or liner was revised, most likely registered as such because the CAL was placed into an existing cup or shell, from which the polyethylene liner was removed. Median follow-up was 4.8 (IQR 2.3–8.2) years. The re-revision rate was 10% (95% CI, 6.6–14) at 5-year follow-up and 12% (95% CI, 8.1–17) at 9-year follow-up (Figure 1). The main reason for re-revision was dislocation (*n* = 19). Loosening of the acetabular component was the reason for re-revision in 3 procedures. In 9 cases at least the acetabular component was revised, and in 11 cases the femoral head and/or liner was revised (Table 4).

**Table 3.** Type and reason for revision hip arthroplasty using a constrained acetabular liner.

Type of revision (%)	*n* = 236
Total revision	21 (10)
Partial revision, at least revision acetabulum	123 (52)
Partial revision, at least revision femur	13 (5.5)
Partial revision, revision femoral head land/or liner	79 (33)
Girdlestone (including spacer)	0 (0.0)
Other	2 (7.2)
Reason for revision^a^ (%)	
Dislocation	183 (81)
Infection	4 (1.9)
Wear	21 (15)
Girdlestone	2 ( 1.0)
Loosening acetabulum	17 (8.1)
Loosening femur	12 (5.8)
Pari-articular ossifications	1 (0.5)
Metal-on-metal bearing	2 (1.0)
Other	25 (17)

a The sum is higher than the total number since >1 reason for revision can be registered.

**Table 4.** Type and reason for re-revision of constrained acetabular liners.

Type of re-revision (%)	*n* = 28
Total revision	3 (10.7)
Partial revision, at least revision acetabulum	9 (32.1)
Partial revision, revision femoral head and/or liner	11 (39)
Girdlestone (including spacer)	3 (10.7)
Other	2 (7.2)
Reasons for re-revision^a^ (%)	
Dislocation	19 (70)
Infection	4 (15)
Wear	3 (14)
Girdlestone	1 ( 4.2)
Loosening acetabulum	3 (12)
Loosening femur	2 ( 8.3)
Other	3 (15)

a The sum is higher than the total number since >1 reason for revision can be registered.

## Discussion

We found a relatively low use of CALs in the Netherlands, considering that only 29 CALs were implanted in primary THA and 236 CALs in revision procedures over 16 years. Primary THAs with a CAL did not require revision after a median follow-up of 5.4 years. The CALs in primary THA were used in patients with high ASA scores in half of the patients, and still resulted in a high survival rate. A few previous studies reported the use of CALs in primary THA. An analysis of the Finnish Arthroplasty Register showed that CALs were used in 373 primary THAs between 2006 and 2017.^8^ The reason for using a CAL in primary THA was not reported. The 8-year survivorship of this group was 94%, with infection as the main reason for revision (10 cases) and only 2 cases of aseptic acetabular loosening. Gill et al.^13^ described a group of 55 patients (mean age 83 years) with 1 mechanical failure of the CAL during a follow-up of 45 months. They reported a high mortality rate (54%), probably because of the high age at implantation.^13^ Another study presented the implantation of CALs in 325 patients with a femoral neck fracture of the hip (mean age 75 years).^14^ They chose a CAL because the incidence of dislocation in this population is relatively high. Only 1 patient had a dislocation of the CAL after a follow-up of 5 years, and 2 patients had loosening of the cup.

CALs implanted in revision hip arthroplasty showed a re-revision rate of 12% after a follow-up of 9 years in our study. A relatively high number of patients died during follow-up of the revision group (38%), which is likely due to the relatively high age at implantation and the fact that a CAL is often implanted as a last resort in fragile patients. The reason for re-revision surgery of the CAL was predominantly dislocation.

The survival of CALs presented in our study is in line with the data presented in a recent meta-analysis into this subject.^7^ Mancino et al.^7^ analysed 37 articles in which 4152 CALs were implanted. CALs were most commonly used in revision THA, usually for the treatment of recurrent dislocation. The re-operation rate was 20.1% at a mean follow-up of 6.9 years. A reoperation rate is potentially higher than a (re-)revision rate, as surgeries not revising (a part of) the prosthesis are also included. Nevertheless, as the review mentions only 3 reoperations for haematoma, 1 for nerve injury and 1 for trochanteric hardware removal, with the rest of the indications requiring a component revision, the difference between reoperation and (re-)revision will be negligible. Dislocation and infection were the most common reasons for re-revision with an incidence of 9.2% and 4.6% respectively. Dislocation of a CAL means by definition mechanical failure, and therefore almost always surgically treated. The type of failure was described in the systematic review, being locking ring failure (36.2%), liner disengagement from the cup (33.6%), loosening of the implant-bone interface (15.9%) and dislocation of the femoral head (9.5%). The survival of CALs decreased with each subsequent constrained liner revision.^15^

Loosening of the acetabular component was the reason for re-revision of the CAL in our study in 3 procedures (12%). The amount of revisions for loosening was low, with 2.9% on average in the previous review including 4152 CALs, of which 426 were placed in primary THA.^7^ The type of fixation of the CAL in revision surgery in our study was most commonly cementless (70%). The cemented cups were partially primarily fixed into the acetabulum and partially cemented into a retained, well-fixed acetabular shell. This last technique has been separately studied, with variable outcomes.^16,17^ Relatively high rates of dissociation of the CAL from the acetabular component were described, with 65% survival for aseptic acetabular loosening at 10 years of follow-up.^17^

The strength of our study is that it represents population-based real world data with a high rate of completeness. To our knowledge, it is the first register study on the use of CALs in primary and revision THA including patient characteristics, survival and reasons for revision. Our study also has limitations due to the nature of a register study. We can only analyse the data that is collected in the register and, for example, are not informed of the reasons for implantation of CALs in primary THA or the type of implant used. We also do not know how many CALs in the revision group were cemented in an existing cup or shell.

In conclusion, data from the Dutch Arthroplasty Register demonstrated a relatively low use of CALs in primary and revision THA. There were no revisions of CALs placed in primary THA at mid-term follow-up. The survival of CALs in revision THA was acceptable, with a re-revision rate of 12% after a follow-up of 9 years. Recurrent dislocation was the main reason for re-revision. These findings are in line with the data previously presented in the literature and justify the use of CALs in specific cases with high risk for dislocation of THA.
